# Cross-cultural adaptation and psychometric properties of the Chinese version of the Orthorexia Beliefs Scale

**DOI:** 10.3389/fpsyt.2026.1729004

**Published:** 2026-02-04

**Authors:** Fan Yan, Xinzhang Sun, Hanqing Zhang

**Affiliations:** 1 Department of Hematology, Henan Provincial People’s Hospital, Zhengzhou, Henan, China; 2 Yangtze University, Health Science Center, Jingzhou, Hubei, China; 3 Faculty of Public Health, Mahidol University, Bangkok, Thailand

**Keywords:** psychometric properties, scale adaptation, validation, orthorexia nervosa, scale

## Abstract

**Background:**

Orthorexia nervosa refers to a pathological obsession with eating “pure” or “healthy” food, and researchers have emphasized the need for psychometrically sound instruments to assess underlying belief systems rather than behavioral outcomes. The Orthorexia Beliefs Scale (OBS) was developed for this purpose, but no validated Chinese version exists.

**Objective:**

This study aimed to translate and culturally adapt the OBS into Chinese and to evaluate its psychometric properties, including reliability and construct validity, in a Chinese nonclinical sample.

**Methods:**

The original OBS was translated following standard forward-backward translation procedures and refined through expert review and pilot testing. A total of 352 Chinese participants completed the questionnaire, which was randomly split into two subsamples (N_1_ = 152 for exploratory factor analysis; N_2_ = 200 for confirmatory factor analysis). Item analysis, internal consistency (Cronbach’s α, McDonald’s ω), content validity (CVI), exploratory factor analysis (EFA), and confirmatory factor analysis (CFA) were conducted.

**Results:**

Item–total correlations ranged from 0.46 to 0.64. Content validity indices (I-CVI) were high across items, and the scale-level S-CVI/Ave was 0.99. The EFA extracted three factors explaining 50.3% of variance, consistent with the original theoretical structure. CFA results showed acceptable model fit: χ²(167) = 265.66, p <.001, χ²/df = 1.59, CFI = 0.949, TLI = 0.942, RMSEA = 0.053 (90% CI [.041,.065], PCLOSE = 0.328). Cronbach’s α and ω were above 0.80 for the total scale and all subscales. Test–retest reliability (n = 30, two-week interval) yielded intraclass correlation coefficients (ICC) above 0.75.

**Conclusions:**

The Chinese version of OBS (OBS-C) demonstrated strong reliability and validity in a nonclinical Chinese sample. It is a psychometrically sound instrument suited for assessing orthorexia-related belief systems in Chinese populations, laying a foundation for future research and interventions on orthorexia tendencies in China.

## 1 Introduction

Orthorexia is originated from Greek ortho created by Bratman (1) which means “right appetite.” In this regard, research shows that the concept of “orthorexia” can be divided into two dimensions: orthorexia nervosa (OrNe) and healthy orthorexia (2). A condition is classified as orthorexia nervosa when an individual’s preoccupation with healthy eating evolves into clinically significant obsessive beliefs accompanied by impaired social functioning, anxiety disorders, or substantial distress. In contrast, healthy orthorexia—while also characterized by persistent attention to dietary health—manifests cognitive-behavioral patterns that do not elicit functional impairment or subjective suffering. Evidence indicates that orthorexia nervosa shows significant associations with compromised overall social functioning and diminished dietary quality, whereas healthy orthorexia correlates positively with scientifically grounded eating behaviors and more structured nutritional knowledge (3). In a landmark international consensus convened in 2022, 47 eating disorder specialists representing 14 countries formally recognized Orthorexia Nervosa (ON) as a distinct mental health disorder. This decision positions ON within the diagnostic taxonomy of Feeding and Eating Disorders (F&ED) as outlined in the Diagnostic and Statistical Manual of Mental Disorders, Fifth Edition (DSM-5) (4). An excessive preoccupation with dietary purity and rigid adherence to “clean” eating patterns may initiate the development of distinct eating disorder phenotypes, frequently generating clinically significant adverse outcomes that paradoxically contradict the purported health objectives. Empirical evidence indicates that Orthorexia Nervosa (ON) can precipitate a range of detrimental health consequences, including nutrition deficiencies and progressive deterioration of social functioning (1).

Orthorexia Nervosa (OrNe) manifests multiple pathophysiological characteristics consistent with established eating disorders including anorexia nervosa (AN), bulimia nervosa, and binge-eating disorder (5). Empirical evidence further demonstrates significant associations between OrNe and both obsessive-compulsive disorder (OCD) and obsessive-compulsive personality disorder. A recent meta-analysis has substantiated a moderate correlation (r = 0.40) between core OCD symptomatology and OrNe clinical presentations, providing important evidence for understanding their comorbid mechanisms and clinical trajectories (6). A study has shown that almost all subtypes of OCD are associated with ON, with “obsession” being the strongest and “checking” being the weakest (7). Indeed, this obsessive foundation might represent the connection between eating disorders, particularly the restrictive subtype of AN—and OrNe. Exploring this link could yield valuable insights into the psychopathology of OrNe and its relation to HeOr (5).

Although the OBQ has been frequently used, it was originally designed to capture general OCD-related beliefs rather than eating-specific cognitions. Consequently, its utility for measuring OrNe is limited. Moreover, the OBQ does not assess OCD symptoms directly but rather underlying beliefs that may contribute to the disorder. As noted by the OCCWG (2003), “any novel scientific approach to a psychological disorder must define its central concepts and processes in a way that allows for measurement and testing” (p. 864). The beliefs reflected in the OBQ are viewed as mechanisms that may contribute to the onset and persistence of OCD, rather than the symptomatic features that meet diagnostic criteria (5).

This study aims to translate and adapt the Orthorexia Beliefs Scale (OBS) (5) into Chinese. The OBS was designed to specifically assess dysfunctional beliefs related to orthorexia, complementing existing measures of Orthorexia Nervosa (OrNe) and Healthy Orthorexia (HeOr). By localizing the scale, we seek to clarify the role of cognitive beliefs in the development and maintenance of orthorexia and provide a theoretical basis for targeted interventions.

## 2 Methods

### 2.1 Original OBS

The Orthorexia Beliefs Scale (OBS) is a measurement tool specifically designed to assess dysfunctional beliefs associated with orthorexia. It comprises three dimensions—Overvaluation of Healthy Eating, Moral Meaning, and Need to Control—with a total of 21 items. Each item is rated on a 4-point Likert scale ranging from 0 (“strongly disagree”) to 3 (“strongly agree”). The scale allows for the calculation of both subscale scores and an overall score (ranging from 0 to 63).

### 2.2 Translation and culture adaptation

With permission from the original developers of the Orthorexia Beliefs Scale (OBS), the translation and cultural adaptation were conducted in strict accordance with established methodological guidelines. To ensure both linguistic precision and cultural relevance, a standardized forward–backward translation procedure was implemented. The cross-cultural adaptation process rigorously followed Brislin’s model, resulting in the finalized Chinese version of the OBS (8–10).

#### 2.2.1 Translation and back-translation procedure

The cross-cultural adaptation of the scale was conducted following Brislin’s classical translation model. Initially, two native Chinese-speaking researchers with advanced English proficiency and background in psychological assessment independently performed forward translation of the original instrument. The resulting two Chinese versions underwent systematic comparison and conceptual harmonization through panel discussions to develop a preliminary consolidated version. Subsequently, two native English-speaking bilingual experts familiar with Chinese cultural context were recruited to perform back-translation using a blinded protocol to maintain translational independence. The back-translated versions were iteratively compared with the original scale through multiple rounds of review, incorporating feedback from the original developers to finalize a pre-test version that ensured semantic equivalence and linguistic appropriateness for the target population.

#### 2.2.2 Cross-culture validation

In June 2025, the preliminary Chinese version (C2) underwent refinement using the Delphi method. Seven experts in the field of eating disorders (each possessing over five years of professional experience, senior professional titles, and master’s degrees or higher) evaluated the items against the original scale, focusing on content relevance, cultural appropriateness, and linguistic clarity. Following expert consensus revisions, the final revised Chinese version (C3) was established.

### 2.3 Participants

Following the Delphi consultation, the scale was finalized with 20 items. Following traditional psychometric guidelines, the required sample size was determined to be 5 to 10 times the number of measurement variables. To ensure analytical robustness, exploratory factor analysis (EFA) required at least 120 participants, while confirmatory factor analysis (CFA) demanded at least 200. Accounting for an anticipated 10% attrition rate, the final target sample size was set at no fewer than 352 valid responses (11, 12).

All participants provided informed consent voluntarily after being fully informed of the study details. Participation was entirely voluntary, with no compensation provided, and the anonymity of participants was strictly protected. Inclusion criteria included being at least 18 years of age, a native Mandarin speaker, and capable of completing the questionnaire independently. Individuals with visual or cognitive impairments were excluded from the study.

### 2.4 Statistical software

Prior to data analysis, the research team manually excluded incomplete responses. Data entry and processing were performed using SPSSAU, JAMOVI version 2.3.28, and AMOS version 23.0.

### 2.5 Reliability analysis

Reliability refers to the degree of stability and consistency with which an instrument measures a construct. Higher reliability indicates reduced measurement error and greater confidence that the observed scores accurately represent the underlying variable (13).

To comprehensively evaluate the scale’s internal consistency and temporal stability, this study computed Cronbach’s alpha, McDonald’s omega, split-half reliability, and test-retest reliability. Cronbach’s alpha was used to assess the internal consistency of items within each dimension, with a value exceeding 0.70 considered indicative of acceptable reliability. McDonald’s omega provided a more accurate estimation of internal consistency, particularly given the scale’s multidimensional structure (13). A comprehensive reliability assessment was performed integrating split-half and test-retest methodologies. The split-half reliability analysis involved partitioning scale items into matched halves using odd-even numbering, with subsequent application of the Spearman-Brown prophecy formula to estimate full-scale reliability. The derived coefficient of 0.812 exceeded the psychometric threshold of 0.80, indicating adequate internal consistency. For temporal stability evaluation, intraclass correlation coefficients (ICC) were calculated through repeated administrations to a cohort of 30 participants at a two-week interval. The analysis demonstrated a Pearson correlation coefficient of 0.987 (p < 0.001) between measurements, substantially surpassing the established test-retest reliability standard of 0.70, with all ICC values exceeding 0.75.

### 2.6 Validity analysis

#### 2.6.1 Exploratory factor analysis

The latent structure of the scale was systematically identified through exploratory factor analysis, employing the principal axis factoring method as the core extraction approach to discern clinically meaningful dimensions. Items with factor loadings greater than 0.40 were retained for construct interpretation. This analytical strategy adheres to contemporary psychometric standards for scale validation in psychological research.

#### 2.6.2 Confirmatory factor analysis

Subsequently, a Confirmatory Factor Analysis (CFA) was performed to validate the factor structure proposed by the preceding EFA. As an application of structural equation modeling (SEM), CFA was employed to test the degree of fit between the theoretical model and the observed data. This analysis served to delineate the paths between the observed variables and their latent constructs, while simultaneously evaluating a suite of model fit indices—including χ²/df, RMR, CFI, RMSEA, GFI, and AGFI—thereby validating the construct validity of the scale.

### 2.7 Item analysis

A comprehensive item analysis was systematically conducted to evaluate the psychometric properties of individual scale components. This methodological approach aimed to identify suboptimal items through rigorous assessment of discrimination indices, homogeneity coefficients, and related measurement metrics, ensuring the instrument’s construct validity and statistical robustness (14). The item-total correlation was calculated for each item against the total scale score with that item excluded, to determine its contribution to the overall measurement. Items demonstrating a correlation coefficient above 0.30 were considered to possess satisfactory representativeness within the overall construct, whereas those with lower correlations were flagged as potentially requiring revision (14).

### 2.8 Ethics statement

The portion of this study involving human subjects has been approved by the Medical Ethics Committee of the Sixth Affiliated Hospital of Kunming Medical University (Ethics Approval Number: 2024kmykdx6f024). The implementation of the study complies with relevant local laws, regulations, and institutional requirements. All participants voluntarily enrolled in this study after signing written informed consent forms.

## 3 Results

### 3.1 Demographics

The study enrolled a total of 352 participants, with 152 included in the exploratory factor analysis and 200 in the confirmatory factor analysis. During the initial data processing phase, all cases containing missing items were manually excluded; consequently, all subsequent analyses were performed on a complete dataset. Participants’ ages ranged from 16 to 66 years, with a mean of 26.50 years (SD = 8.43; median = 23), indicating a sample predominantly composed of young and middle‐aged adults. Regarding gender distribution, 234 participants (66.5%) were female and 118 (33.5%) were male.

### 3.2 Scale translation and cross-cultural adaptation

The Chinese version was developed through forward–back translation, with careful consideration of semantics, idiomatic usage, and cultural relevance. Discrepancies were resolved by comparing the translations with the original scale, and the finalized draft was approved by both researchers and linguists. During Delphi review, the item *“Thinking about eating unhealthy foods means that I actually want to eat them”* was excluded as experts judged it inappropriate for retention.

### 3.3 Reliability

#### 3.3.1 Cronbach’s alpha and omega coefficient

The reliability analysis indicated that the overall scale demonstrated excellent internal consistency, with a Cronbach’s α of 0.918 and a McDonald’s ω of 0.918. The mean score was 1.63 (SD = 0.554). Item-level reliability analysis showed that the deletion of any individual item did not lead to a notable improvement in reliability, with Cronbach’s α values ranging from 0.910 to 0.920 and McDonald’s ω ranging from 0.911 to 0.920. These findings suggest that all 20 items contributed meaningfully to the overall reliability of the scale, and no item was identified as redundant or problematic. The three subscales demonstrated good internal consistency. Cronbach’s α and McDonald’s ω were 0.839 and 0.842 for Overvaluation of Healthy Eating (OHE), 0.877 and 0.880 for Moral Meaning (MM), and 0.840 and 0.841 for Need to Control (NC), respectively.

#### 3.3.2 Split-half reliability

The split-half reliability of the scale was assessed using the Spearman-Brown formula. The resulting reliability coefficient was 0.812, exceeding the commonly accepted psychometric threshold of 0.80, which indicates satisfactory internal consistency of the instrument.

#### 3.3.3 Test–retest reliability

To evaluate the temporal stability of the scale, a subset of 30 participants was retested two weeks after the initial administration. The test–retest reliability, calculated using the intraclass correlation coefficient (ICC), was 0.987, demonstrating a high degree of measurement consistency over time.

### 3.4 Validity

#### 3.4.1 Content validity analysis

The content validity of the Chinese version of the OBS was assessed by seven experts using a 4-point relevance scale. Items rated 3 or 4 were considered relevant. The item-level content validity indices (I-CVI) ranged from 0.71 to 1.00. Nineteen of the twenty items achieved universal agreement among experts (S-CVI/UA = 0.95), and the average scale-level CVI (S-CVI/Ave) was 0.99.

#### 3.4.2 EFA

The KMO coefficient was 0.881, exceeding the 0.6 threshold and indicating the adequacy of the data for factor analysis. Bartlett’s test of sphericity was also significant (p < 0.05), confirming the suitability of the data. Using Principal Axis Factoring with Promax rotation, the analysis extracted three latent factors from the 20 items. These factors captured the major variance structure with substantial loadings. Specifically, Factor 1 included items Q1–Q8 (loadings = 0.555–0.813), Factor 2 comprised items Q9–Q15 (loadings = 0.637–0.798), and Factor 3 covered items Q16–Q20 (loadings = 0.651–0.787). The detailed factor loadings are presented in **Table 1**. Sample size: 152.

**Table 1:** Factor loadings of each item in the Chineseversion OBS.

Exploratory factor analysis				
Factor loadings				
Items	Factor			
	1	2	3	Uniqueness
Q9	0.779			0.449
Q13	0.731			0.465
Q10	0.72			0.472
Q12	0.698			0.487
Q15	0.69			0.485
Q11	0.689			0.485
Q14	0.631			0.459
Q3		0.776		0.5
Q2		0.699		0.545
Q8		0.644		0.54
Q4		0.644		0.52
Q6		0.628		0.529
Q5		0.616		0.532
Q1		0.565		0.54
Q7		0.542		0.537
Q17			0.756	0.461
Q18			0.706	0.493
Q19			0.678	0.513
Q20			0.663	0.494
Q16			0.634	0.44

'Principal axis factoring' extraction method was used in combination with a 'oblimin' rotation.

#### 3.4.3 CFA

**Figure 1:**
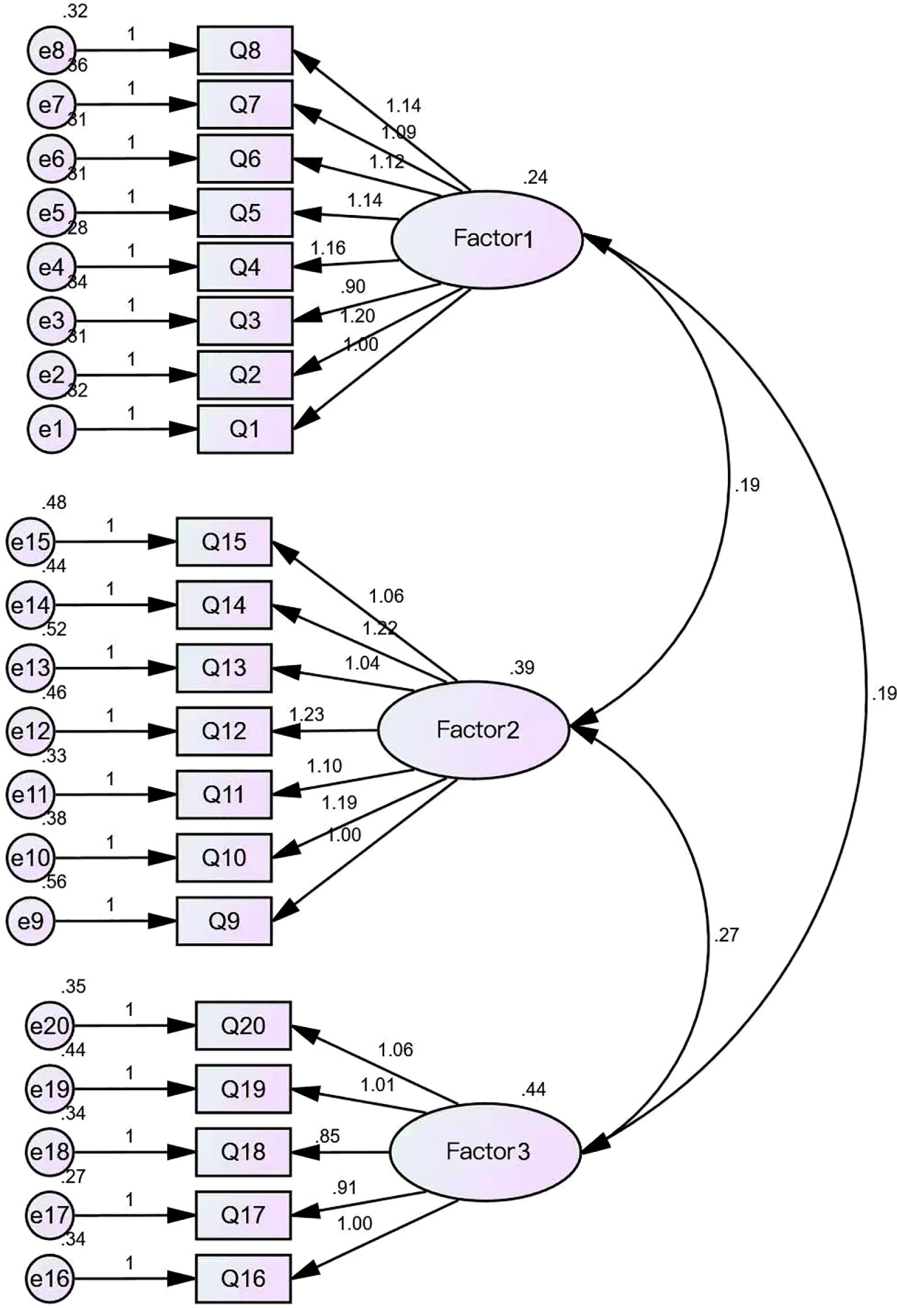
CFA Path Diagram of OBS-C.

Confirmatory factor analysis (CFA) was conducted using Amos 24.0 software on the Chinese version of the OBS scale to examine its measurement structure. The standardized three-factor model (**Figure 1**; N = 200) demonstrated good overall fit with the data. The chi-square test yielded significant results, χ² (167) = 265.66, p <.001. However, the chi-square/degrees of freedom ratio (χ²/df = 1.59) fell below the recommended threshold of 2.0, indicating the model’s good parsimony. The comparative fit index (CFI = 0.949) and Tucker–Lewis index (TLI = 0.942) both exceeded the 0.90 threshold, indicating good incremental fit. The root mean square error of approximation (RMSEA) was.053, with a 90% confidence interval [0.041, 0.065], below the recommended upper limit of 0.08. Additionally, PCLOSE = 0.328 (> 0.05), indicating good model-data fit with no significant approximation error. The noncentral parameter (NCP) was estimated at 98.664 (90% CI [58.181, 147.073]), further indicating minimal deviation from the null hypothesis. The Goodness-of-Fit Index (GFI = 0.888) and Adjusted Goodness-of-Fit Index (AGFI = 0.859) approached recommended values, further supporting model adequacy. In summary, the three-factor model adequately reflects the observed data structure, indicating that the Chinese version of the OBS possesses good construct validity.

### 3.5 Item analysis

All items demonstrated positive item-total correlations. Correlation coefficients ranged from 0.46 to 0.64. Item Q3 exhibited the lowest correlation (r = 0.47), while item Q14 showed the highest correlation (r = 0.64). None of the item correlations fell below the threshold of 0.30.

## 4 Discussion

In accordance with standardized procedures for cross-cultural translation and adaptation of measurement instruments, and incorporating evaluations from multiple domain experts, this study systematically adapted the original Obsessive Beauty Scale (OBS) into Chinese (OBS-C). Psychometric evaluations demonstrated that the OBS-C exhibits satisfactory item discrimination, internal consistency, test-retest reliability, and construct validity, confirming its stable and reliable measurement properties. Exploratory factor analysis extracted three latent dimensions, and subsequent confirmatory factor analysis validated this structure. The model fit indices indicated an acceptable fit, supporting both the theoretical plausibility and empirical validity of the three-factor model. These findings collectively affirm OBS-C’s applicability and measurement efficacy within the target population.

### 4.1 Psychometric properties and model fit results

This study conducted a systematic psychometric evaluation to validate the measurement properties of the Chinese version of the OBS scale (OBS-C) across multiple dimensions. The detailed results are presented as follows:

Item analysis revealed that all item-total correlations ranged from 0.46 to 0.64, exceeding the conventional threshold of 0.30. This indicates that all items effectively represent the target construct and demonstrate satisfactory discriminant validity. The concentrated distribution of correlation coefficients further reflects strong internal consistency within the scale, with no significant evidence of measurement redundancy.

Regarding reliability, the scale demonstrated excellent internal consistency. Furthermore, test-retest reliability was assessed with a two-week interval among 30 participants, yielding a reliability coefficient of 0.987 (p < 0.001). This indicates high temporal stability of the measurement. Although this coefficient is notably high, it is recommended that future studies validate this finding with larger sample sizes and extended retest intervals to further establish stability over time.

In terms of construct validity, exploratory factor analysis identified three common factors with eigenvalues greater than 1, collectively accounting for 50.3% of the total variance. The variance contributions of the three factors were 18.7%, 18.0%, and 13.5%, respectively, demonstrating strong consistency with the theoretical framework of the original scale and indicating successful retention of the core dimensional structure in the Chinese version.

Confirmatory factor analysis further supported the structural validity of the model. The fit indices indicated good model-data agreement: χ²/df = 1.59 (< 3.0), CFI = 0.949, TLI = 0.942 (both > 0.90), and RMSEA = 0.053 (90% CI: 0.041–0.065, < 0.08). Although the GFI (0.888) and AGFI (0.859) were slightly below the ideal threshold of 0.90, these values remain within acceptable ranges considering sample characteristics and model complexity. Comprehensively, the three-factor model demonstrates satisfactory fit and theoretical consistency.

### 4.2 Significance of study

The translation of the Orthodox Belief Scale (OBS) into Chinese represents a crucial step in advancing research on orthodox eating tendencies within the Chinese context. Most existing orthodox eating scales originate from Western cultures, whose health concepts, dietary norms, and social value orientations differ significantly from Chinese culture. Direct use of the original English-language scales may introduce measurement bias, resulting in research findings that lack cultural appropriateness and generalizability. The development of the Chinese OBS enables researchers to more accurately assess the cognitive beliefs and irrational attitudes underlying orthorexia behaviors within a framework aligned with Chinese cultural and linguistic characteristics. This tool not only facilitates cross-cultural comparative studies but also provides a reliable basis for clinical screening and epidemiological investigations, thereby promoting early identification and intervention of orthorexia tendencies in public health settings. Thus, the localized translation and validation of the OBS fills a gap in eating disorder measurement tools within China and provides a measurement instrument for future exploration of dietary attitudes, health beliefs, and related psychological mechanisms.

## 5 Limitations

The design and application of this scale strictly adhere to modern psychometric principles, as it does not establish nor recommend the implementation of any diagnostic or screening cutoff scores. Its primary function is to serve as a quantitative measurement tool for assessing relevant cognitive beliefs in research and clinical evaluation contexts, rather than providing categorical diagnostic decisions. The original development literature explicitly emphasizes that the instrument’s purpose was not to create a new questionnaire for evaluating orthorexia tendencies, but rather to precisely measure the latent cognitive processes associated with orthorexia concerns. Based on its measurement characteristics, the OBS should not be regarded as a replacement for existing orthorexia assessment tools but rather should be understood as a complementary instrument with distinct measurement foci and theoretical constructs. Developed within a continuous measurement framework, this psychological instrument was designed to preserve the complete dimensional information of measurement data, as opposed to artificially dichotomizing continuous spectrum characteristics into positive/negative categories through fixed cutoff points. This design philosophy is grounded in the fundamental principles of classical test theory regarding the superiority of continuous measurement over categorical classification. This approach both preserves data integrity and optimizes statistical power, thereby aligning with contemporary psychometric requirements for precise quantification and scientific assessment.

## 6 Conclusion

This study systematically conducted the translation, cross-cultural adaptation, and psychometric validation of the Chinese version of the Orthorexia Beliefs Scale (OBS-C). Employing a rigorous forward-backward translation protocol, Delphi expert consultation, and large-scale empirical testing, the OBS-C demonstrated strong reliability, robust internal consistency, and satisfactory construct validity. Both exploratory and confirmatory factor analyses consistently supported a three-dimensional structure—comprising Overvaluation of Healthy Eating, Moral Meaning, and Need for Control—that aligns closely with the original theoretical framework. The validated Chinese version provides a reliable and culturally appropriate instrument for assessing orthorexia-related belief systems in Chinese populations. Its availability establishes a foundation for future cross-cultural comparative studies, clinical screening initiatives, and investigations into the cognitive mechanisms underlying both orthorexia nervosa and healthy orthorexia. In summary, the development of the OBS-C addresses a critical methodological gap in the assessment of orthorexia within non-Western contexts and provides a validated tool for subsequent epidemiological surveillance and intervention research in China.

## Data Availability

The raw data supporting the conclusions of this article will be made available by the authors, without undue reservation.
