# Supplementary material for: Cross-cultural adaptation and psychometric properties of the Chinese version of the Orthorexia Beliefs Scale
Source: Front Psychiatry. 2026 Feb 4;17:1729004. doi: 10.3389/fpsyt.2026.1729004 (PMC12913363; doi:10.3389/fpsyt.2026.1729004)
Supplement: Supplementary file 1 [file Table1.docx]

**Independent Samples T-Test**

| Independent Samples T-Test | | | | | | | | | |
| --- | --- | --- | --- | --- | --- | --- | --- | --- | --- |
|  | |  | | **Statistic** | | **df** | | **p** | |
| total scores |  | Student's t |  | -1.30 |  | 350 |  | 0.196 |  |
| Note. Hₐ μ _1_ ≠ μ _2_ | | | | | | | | | |
|  | | | | | | | | | |

| Group Descriptives | | | | | | | | | | | | | |
| --- | --- | --- | --- | --- | --- | --- | --- | --- | --- | --- | --- | --- | --- |
|  | | **Group** | | **N** | | **Mean** | | **Median** | | **SD** | | **SE** | |
| total scores |  | 1 |  | 234 |  | 48.0 |  | 49.0 |  | 13.4 |  | 0.877 |  |
|  | | 2 |  | 118 |  | 50.0 |  | 51.5 |  | 13.9 |  | 1.28 |  |
|  | | | | | | | | | | | | | |

*Female：1 Male：2*

**Table S1. Descriptive statistics of OBS total scores for the total sample and by gender.**
