# Supplementary material for: Cross-cultural adaptation and psychometric properties of the Chinese version of the Orthorexia Beliefs Scale
Source: Front Psychiatry. 2026 Feb 4;17:1729004. doi: 10.3389/fpsyt.2026.1729004 (PMC12913363; doi:10.3389/fpsyt.2026.1729004)
Supplement: Supplementary file 2 [file Table2.docx]

**One-Way ANOVA**

| One-Way ANOVA (Welch's) | | | | | | | | | |
| --- | --- | --- | --- | --- | --- | --- | --- | --- | --- |
|  | | **F** | | **df1** | | **df2** | | **p** | |
| total scores |  | 9.05 |  | 2 |  | 59.1 |  | < .001 |  |
|  | | | | | | | | | |

| Group Descriptives | | | | | | | | | | | |
| --- | --- | --- | --- | --- | --- | --- | --- | --- | --- | --- | --- |
|  | | **Age-group** | | **N** | | **Mean** | | **SD** | | **SE** | |
| total scores |  | 1 |  | 223 |  | 46.6 |  | 14.4 |  | 0.962 |  |
|  |  | 2 |  | 108 |  | 51.7 |  | 11.6 |  | 1.117 |  |
|  |  | 3 |  | 21 |  | 54.7 |  | 10.0 |  | 2.192 |  |
|  | | | | | | | | | | | |

*1：18-25*

*2：26-40*

*3：＞40*

**Table S2. Descriptive statistics of OBS total scores for the total sample and age group.**
